# Annotations

**Published:** 1907-06-22

**Authors:** 


### Annotations.

### Delayed Chloroform Poisoning.

This condition affords another example of the value of carefully recorded clinical observations. It is but a few years since the first cases were described, and already a considerable body of literature dealing with the subject has come into existence. In this country the pioneer work in connection with delayed chloroform poisoning lies to the credit of Dr. Leonard Guthrie, and valuable contributions have more recently been made by Mr. Harold Stiles and Dr. Stuart McDonald, of Edinburgh. Concerning the true explanation of the condition much difference of opinion has existed, and some surgeons have refused to believe that the symptoms were consequences of the anæsthetic. It now seems to be fairly well established that the essential and underlying fact is an acute acid intoxication, and that post-mortem examination reveals extensive fatty changes in the liver and other organs. Therefore it is not surprising to find that a survey of the recorded cases shows that it is in conditions associated with fatty liver that this form of poisoning is apt to develop. These Dr. Guthrie defines as sepsis, acute and chronic diabetes, rickets and infantile paralysis which have been treated by overfattening and under-exercise, cyclical vomiting with acetonuria, starvation, and probably, also, alcoholism. Hence he urges that an operation should be delayed when there is a history or evidence of one or other of such states; and also when a child has recently vomited without apparent cause. Patients with suspected fatty liver should be kept for several days prior to operation on a diet restricted in fats, and bicarbonate and citrate of sodium should be given. As starvation may give rise to acute acetonæmia, nutrient enemata should be ordered both two hours before and immediately after an operation. When symptoms of acid intoxication appear subsequent to operation, the treatment should be by venesection, saline transfusion, and enemata of solution of sodium bicarbonate.

### The Society for the Study of Disease in Children.

For several years this Society has adopted the custom of holding one meeting in each session in a provincial centre, and invariably with excellent results. Not only are local interest and activity quickened, but the London members get an opportunity of appreciating the excellent arrangements and opportunities which exist apart from the metropolis, and the particular work of the Society is definitely encouraged. This year Bedford was selected as the meeting-place, and the event has more than justified the choice. By kind permission of the governing authorities, the County Hospital was thrown open to the Society, and an excellent and interesting programme was arranged. The visitors, too, had an opportunity of seeing the various departments of the hospital, and these certainly well repaid inspection. The earlier part of the programme included a demonstration of clinical cases, microscopical specimens and skiagrams. Among the cases shown was a typical example of achondroplasia, a case of coloboma of each iris and choroid, an instance of congenital dilatation of the colon, a case of unilateral hypertrophy, and a number of patients with cardiac disease. Later in the day the Society was occupied with a discussion on Acute Rheumatism in Childhood, over which Dr. R. H. Coombs presided, and in which many members took part. Thereafter an excellent paper on Cerebro-spinal Meningitis was read by Dr. Cautley, followed by an interesting communication on halting and stumbling in children by Mr. Tubby. Mr. Henry Skelding also read a note on a case of bilharzia hæmatobia in a boy, and showed specimens of the ova under the microscope. In the evening a dinner was given in the Swan Hotel under the chairmanship of Mr. Clement Lucas, when cordial votes of thanks were passed to the members of the local profession to whose efforts so much of the success of the meeting was due. Especial recognition must be extended to Mr. Jaffrey and to Mr. Skelding, who were immediately responsible for all the arrangements; these were excellent, and highly appreciated by all the visitors.

### Physically Defective Children.

In his speech at the recent meeting of the After-care Committee for Blind, Deaf, and Cripple Children the Bishop of Stepney quoted some figures which are of much interest, and which show the varied responsibilities of the public educational authorities. Thus under the authority of the London County Council, it appears, there are 22 schools for cripples alone, with 1,800 children in attendance; there are 300 children in special schools for the blind, and 620 in special schools for the deaf, and it is estimated that in a very short time as many as 300 children will be leaving these schools every year. When the child's school days are over the authority of the education department ceases, and the charity in the interests of which the Bishop quoted these figures then intervenes with the object of teaching the child some useful employment. We are glad to see that the Bishop made a public acknowledgment of the kindness and sympathy with which these unfortunate children are usually treated, even in the homes of the poorest classes. This statement will be fully confirmed by every medical practitioner whose lot it is to work in the crowded districts of our big cities. But a real affection and sympathy sometimes lead to the absence of systematic training and discipline, and thus many of these partially disabled children grow up without any of the capacity for self-control which is so necessary to efficiency in life. The educational authorities and the After-care Committee are co-operating to correct this possibility, and as their work is ordered on sound physiological lines it has every promise of success and permanency. Mentally defective children form an even more anxious class, and for many of these little or nothing can be done. Even when an institutional training is available the mother refuses to be separated from her child, and it is difficult to blame her.

